# Cut-Off Points for Mild, Moderate, and Severe Pain on the Numeric Rating Scale for Pain in Patients with Chronic Musculoskeletal Pain: Variability and Influence of Sex and Catastrophizing

**DOI:** 10.3389/fpsyg.2016.01466

**Published:** 2016-09-30

**Authors:** Anne M. Boonstra, Roy E. Stewart, Albère J. A. Köke, René F. A. Oosterwijk, Jeannette L. Swaan, Karlein M. G. Schreurs, Henrica R. Schiphorst Preuper

**Affiliations:** ^1^‘Revalidatie Friesland’ Centre for RehabilitationBeetsterzwaag, Netherlands; ^2^Department of Health Sciences, Community and Occupational Medicine, University Medical Centre Groningen, University of GroningenGroningen, Netherlands; ^3^Adelante Centre of Expertise in Rehabilitation and AudiologyHoensbroek, Netherlands; ^4^Department of Rehabilitation Medicine, CAPHRI Research School, Maastricht UniversityMaastricht, Netherlands; ^5^Faculty of Health and Technology, Zuyd University for Applied SciencesHeerlen, Netherlands; ^6^Department of Rehabilitation Medicine, MGG Medical Centre Alkmaar and Gemini Hospital Den HelderAlkmaar, Netherlands; ^7^Rijndam Rehabilitation InstituteRotterdam, Netherlands; ^8^Roessingh Research and Development, University of TwenteEnschede, Netherlands; ^9^Department of Rehabilitation, Centre for Rehabilitation, University Medical Centre Groningen, University of GroningenGroningen, Netherlands

**Keywords:** musculoskeletal pain, numeric rating scale, pain interference, classification, chronic pain

## Abstract

**Objectives:** The 0–10 Numeric Rating Scale (NRS) is often used in pain management. The aims of our study were to determine the cut-off points for mild, moderate, and severe pain in terms of pain-related interference with functioning in patients with chronic musculoskeletal pain, to measure the variability of the optimal cut-off points, and to determine the influence of patients’ catastrophizing and their sex on these cut-off points.

**Methods:** 2854 patients were included. Pain was assessed by the NRS, functioning by the Pain Disability Index (PDI) and catastrophizing by the Pain Catastrophizing Scale (PCS). Cut-off point schemes were tested using ANOVAs with and without using the PSC scores or sex as co-variates and with the interaction between CP scheme and PCS score and sex, respectively. The variability of the optimal cut-off point schemes was quantified using bootstrapping procedure.

**Results and conclusion:** The study showed that NRS scores ≤ 5 correspond to mild, scores of 6–7 to moderate and scores ≥8 to severe pain in terms of pain-related interference with functioning. Bootstrapping analysis identified this optimal NRS cut-off point scheme in 90% of the bootstrapping samples. The interpretation of the NRS is independent of sex, but seems to depend on catastrophizing. In patients with high catastrophizing tendency, the optimal cut-off point scheme equals that for the total study sample, but in patients with a low catastrophizing tendency, NRS scores ≤ 3 correspond to mild, scores of 4–6 to moderate and scores ≥7 to severe pain in terms of interference with functioning. In these optimal cut-off schemes, NRS scores of 4 and 5 correspond to moderate interference with functioning for patients with low catastrophizing tendency and to mild interference for patients with high catastrophizing tendency. Theoretically one would therefore expect that among the patients with NRS scores 4 and 5 there would be a higher average PDI score for those with low catastrophizing than for those with high catastrophizing. However, we found the opposite. The fact that we did not find the same optimal CP scheme in the subgroups with lower and higher catastrophizing tendency may be due to chance variability.

## Introduction

Assessment of pain intensity is considered one of the core outcome domains in clinical pain research (Dworkin et al., 2005), and is thus very commonly applied. The Numeric Rating Scale (NRS) is regarded as one of the best single-item methods available to estimate the intensity of pain (Jensen et al., 1999; Breivik et al., 2000). The NRS assesses pain intensity using a 0–10 ranking scale with 0 representing “no pain” and 10 “unbearable pain” or comparable statement. Clinicians, including psychologists, often use the categories of mild, moderate, and severe to simplify communication between patients and health care professionals. However, translating continuous measures such as NRS into discrete categories is not straightforward. Simply dividing an NRS into mild, moderate, and severe pain by dividing the scale into three equal parts is not a valid method (Serlin et al., 1995). Serlin et al. (1995) tried to solve this problem by correlating pain intensity to the level of interference of the pain with the daily functioning of patients with pain due to cancer, using a specific statistical technique, i.e., estimating how much of the variance in pain-related disability can be explained by different possible pain intensity classifications. Their statistical approach has been repeated for the same patient population, i.e., cancer patients (Paul et al., 2005) as well as being applied to other patient populations (e.g., Zelman et al., 2005; Hirschfeld and Zernikow, 2013; Oldenmenger et al., 2013; Boonstra et al., 2014). Results from the literature (Hirschfeld and Zernikow, 2013; Oldenmenger et al., 2013) show that the cut-off between mild and moderate pain, in terms of pain-related interference with functioning, is mostly placed between 3 and 4, and the cut-off between moderate and severe pain between 6 and 8. The differences may be caused by differences in study samples, pain definitions, and/or measures of functioning. Difference in diagnoses is generally accepted as one of the main causes of differences in cut-off points between studies (Zelman et al., 2003), while differences between study samples may also be explained by chance variation (Hirschfeld and Zernikow, 2013).

An unresolved issue is the influence of psychological factors on cut-off points. Catastrophizing (expecting or worrying about major negative consequences from a situation, even one of minor importance) is associated with pain severity and disability in patients with several chronic pain conditions (Wertli et al., 2014a,b). Another issue is the influence of the patient's sex on the cut-off points. There are clear, though incompletely understood, differences in pain perception between men and women (Rollman and Lautenbacher, 2001; Racine et al., 2012). Only Fejer et al. (2005) have studied the association between sex and the cut-off points for interference with functioning in individuals with neck pain, and found a small difference between male and female patients.

Most studies have classified pain intensity using the statistical method described by Serlin et al. (1995) to estimate how much of the variance in pain-related disability can be explained by different possible pain intensity classifications. The cut-off point scheme explaining the highest proportion of the variance is then chosen as the optimal scheme. Although this method may have shortcomings, its use facilitates comparisons between studies. Hirschfeld and Zernikow (2013) used a bootstrap resampling procedure and found a very large variability in the cut-off points in their sample of children and adolescents with chronic pain. They recommended that studies to define cut-off points include measures of variability for the optimal cut-off points.

The aims of the present study were to determine the optimal cut-off points for mild, moderate, and severe pain in terms of pain-related interference with functioning for patients with chronic musculoskeletal pain, as well as to measure the variability of the optimal cut-off points, and to determine the association between these cut-off points and patients' catastrophizing tendency and their sex.

## Materials and methods

### Patients

The patients included in the study participated in a nationwide survey of patients with musculoskeletal pain, who were referred or admitted to rehabilitation treatment in one of the cooperating rehabilitation centers. The patients were included when they first consulted their rehabilitation physician or started multidisciplinary inpatient or outpatient rehabilitation treatment. The study included patients from five rehabilitation centers, each with one (rehabilitation centers a, b, e), two (rehabilitation center d), or five (rehabilitation center c) treatment sites in the Netherlands. Some of these centers were departments of a university or general hospital, others were stand-alone rehabilitation centers. The centers are located in different parts of the Netherlands, with patients from rural or semi-industrialized areas, living in villages or medium-sized to large towns and cities. Patients were included between the early months of 2012 and mid-2014; the exact time of inclusion differed between the participating rehabilitation centers. Inclusion criteria were: age over 18 years and having had musculoskeletal pain for longer than 3 months. Exclusion criteria were inability to understand Dutch, current major psychiatric disorder (active psychosis, severe depression with risk of suicide attempt, addiction, etc.), unwillingness to provide data for research purposes, a score of “no pain” or missing data on the NRS and more than 3 missing values on the Pain Disability Index (PDI-DV, see measurements).

#### Ethics statement

All procedures followed were in accordance with the ethical standards of the responsible committee on human experimentation (institutional and national) and with the Helsinki Declaration of 1975, as revised in 2000. The data were collected in a setting of usual care, in order to measure the outcome of the treatment. The patients were asked to indicate if they did not allow their anonymous data to be used for the nationwide survey and/or for scientific studies. Because the data were collected during usual care, no approval of a Medical Ethics Committee was needed.

### Study design

Cross-sectional study in the context of care as usual.

### Measurements

#### Characteristics of the sample

The following background characteristics were assessed: age, sex, marital status, duration of current pain period, and localization of pain (mainly back pain, neck pain including cervicobrachialgia, widespread pain including fibromyalgia, pain in an extremity including shoulder pain, other).

#### Pain intensity and catastrophizing

The NRS for pain is an 11-point numeric rating scale, with 0 representing “no pain” and 10 “unbearable pain.” The patients were asked to assign a number to their average pain in the last week. We decided to ask the patients to report their average pain, as two studies found no differences in the cut-off point schemes of the NRS for average and worst pain (Paul et al., 2005; Zelman et al., 2005) and one study found only a small difference (Fejer et al., 2005). Zelman et al. (2003) also preferred the average pain measure for cut-off point derivation, because in their view average pain better reflects the experiences regarding the interference of pain with daily activities and is more stable than worst pain.

Catastrophizing was evaluated by the Pain Catastrophizing Scale (PCS; Osman et al., 1997). In this questionnaire the patients were asked to reflect on past painful experiences and indicate the degree to which they experienced each of 13 thoughts or feelings when in pain, on a 5-point scale from 0 (not at all) to 4 (all the time). Three or less missing values per patient were replaced by the mean score of the other values. Pain catastrophizing affects how individuals experience pain: ruminating about their pain (e.g., “I can't stop thinking about how much it hurts”), magnifying their pain (e.g., “I'm afraid that something serious might happen”), or feeling helpless to manage their pain (e.g., “There is nothing I can do to reduce the intensity of my pain”). A higher score means greater dominance of the subscale. The total score on the PCS was used in the analyses.

#### Functioning

Interference with functioning was assessed with the Pain Disability Index, Dutch Version (PDI-DV; Soer et al., 2013). The PDI is a 7-item questionnaire to investigate the magnitude of self-reported disability in different situations such as work, leisure time, self-care, and social activities. Each item is scored on an 11-item numeric rating scale in which 0 means no disability and 10 maximum disability. Three or less missing values per patient were replaced by the mean score of the other values. A higher score means greater disability and therefore greater interference with functioning.

#### Procedure

All data were collected prior to the start or in the first 2 weeks of the rehabilitation program.

### Statistical analysis

Descriptive statistics were used to analyze the characteristics of the study sample. Marital status was dichotomized into living alone vs. being married or living with a partner.

#### Cut-off points on the NRS in relation to interference of pain with functioning

Each patient's pain intensity rating on the NRS was classified into three categories, viz. mild, moderate, and severe interference. We analyzed all 28 possible classification schemes, ranging from 2,3 to 8,9. The cut-off points in these classification schemes were named after the upper values for the mild and moderate categories, in accordance with Serlin et al. (1995). For example, a 3,7 CP scheme means that the first category ranges from 1 to 3, the second from 4 to 7 and the third from 8 to 10. The first number, i.e., 3, is thus the upper value of the mild category and the second number, i.e., 7, the upper value of the moderate category. Other examples of schemes are: the 2,5 CP scheme with 1–2 classified as mild, 3–5 as moderate, and 6–10 as severe; the 3,5 CP scheme with 1–3 classified as mild, 4–5 as moderate, and 6–10 as severe; the 5,6 CP scheme with 1–5 classified as mild, 6 as moderate, and 7–10 as severe; and the 5,8 CP scheme with 1–5 classified as mild, 6–8 as moderate, and 9–10 as severe.

In order to determine which CP scheme best distinguished between mild, moderate and severe pain, we used the method introduced by Serlin et al. (1995). We conducted one-way ANOVAs (using the Generalized Linear Model in SPSS, version 22) for each of the 28 classification schemes, using NRS scores recoded as 1, 2, or 3 (depending on the CP scheme) as the independent variable and PDI-DV scores as the dependent variables. A significant *F*-value of the CP scheme indicated that there were significant differences between the three pain severity categories in terms of pain-related interference. In accordance with Serlin et al. (1995), we interpreted the highest *F*-value as indicating the classification scheme that maximized the differences between the groups and was therefore the most useful for distinguishing between mild, moderate, and severe pain-related interference.

The variability of the optimal CP scheme was quantified using a bootstrap resampling procedure (STATA, version 13.1). In this procedure the distribution is estimated using the information based on a number of resamples from the total sample. One thousand (1000) repetitions of samples of the patients were used to yield sufficiently stable estimates for the variability of the optimal cut-off points. The optimal CP scheme for each of the 1000 randomly chosen samples was determined, using the above-mentioned method introduced by Serlin et al. (1995).

#### Association of catastrophizing and patient's sex with the cut-off points for mild, moderate, and severe pain in terms of pain-related interference with functioning

The associations between the cut-off point schemes and the patients' catastrophizing tendency and sex were determined by once again conducting ANOVAs (using the Generalized Linear Model in SPSS, version 22) for each of the 28 CP schemes. In the two series of additional analyses (i.e., with PCS total score and sex), the NRS (recoded as 1–3) was again used as the independent variable and the PDI-DV score as the dependent variable, while the total score on the PSC and the patient's sex were respectively included as co-variates, as was the interaction between CP scheme and PCS score and sex, respectively. In view of the results of the analyses with the PCS score, we decided to conduct separate analyses, firstly for the patients with a PCS score equal to or lower than the median of the PCS scores and the patients with a PCS score higher than the median of the PCS scores (dividing the population into two groups by the median split method), and secondly for patients in the lower and higher quartiles and the middle group of scores (dividing the population into three groups by the quartile split method). In total, therefore, 7 times 28 (196) ANOVAs were conducted. Again, the *F*-values of the CP schemes were used to determine which scheme fitted best. In these two (median split method) and three (quartile split method) patient subgroups we also conducted the bootstrap resampling procedure described above.

## Results

A total of 2854 patients enrolled in the study. Patient characteristics are presented in Table 1. The results of the ANOVAs for the total population are presented in Table 2, which lists only the mid-range of CP schemes. The *F*-values of the CP schemes not presented here were lower than the *F*-value with ranking 6 as indicated in Table 2. The 5,7 CP scheme had the highest *F*-value, indicating that this scheme provided the best fit for distinguishing pain into three categories, i.e., mild, moderate, or severe pain, in terms of interference with functioning. This means that an NRS score in the 1–5 range corresponds to mild interference with functioning, while scores of 6 and 7 represent moderate interference and a score in the 8–10 range corresponds to severe interference with functioning. The mean PDI scores of the patients with NRS scores in the range of 1–5, 6–7, and 8–10 were 30.3 (*SD* 11.8), 39.7 (*SD* 10.6), and 45.4 (11.5), respectively.

**Table 1:** **Characteristics of patients with chronic musculoskeletal pain, Pain Disability Index (PDI) scores, numeric rating scale (NRS) for pain scores and Pain Catastrophizing Scale (PCS) scores, for total sample (***n*** = 2854) and for each rehabilitation center (***n*** total = a:435, b:539, c:840, d:683, e: 357)**.

	**All patients**		**Rehab center a**		**Rehab center b**		**Rehab center c**		**Rehab center d**		**Rehab center e**	
	***n***		***n***		***n***		***n***		***n***		***n***	
**CHARACTERISTICS**												
Age (years, mean (*SD*))	2794	43 (12.5)	435	44 (11.5)	539	42 (12.2)	840	43 (12.8)	679	43 (13.1)	301	43 (12.2)
Sex (% male)	2789	28	431	30	539	24	840	31	678	28	301	29
Marital status (% single)	2746	30	434	29	535	35	817	29	674	30	286	30
Work (%)	2657		319		531		835		673		299	
• Employed or self-employed		51		47		62		50		50		43
• Student		4		2		6		5		4		4
• Without work, or homemaker		29		28		24		28		31		40
• Retired		4		4		2		4		5		4
• Other/mixed		12		19		7		14		10		10
**Location of pain (%)**	2854		435		539		840		683		357	
• Widespread pain		18		10		36		26		7		
• Neck pain		8		1		21		13		2		
• Back pain		18		4		24		35		10		
• Pain in extremity		7		0		9		18		2		
• Others		4		1		4		7		4		
• Unknown		45		84		6		1		75		100
**Duration of complaints (%)**	2503		154		533		836		679		301	
• 3–6 months		5		1		8		4		5		3
• 6–12 months		11		9		12		12		12		10
• 1–2 years		20		20		18		24		18		19
• 2–5 years		25		19		25		23		27		27
•>5 years		39		52		37		38		38		42
**FUNCTIONING**												
**PDI** (mean, *SD*)	2854	39 (12.6)	435	37 (12.7)	539	41 (11.8)	840	37 (12.7)	683	36 (13.2)	357	40 (12.4)
**PAIN**												
**NRS** (median, quartiles)	2854	7 (5–8)	435	6 (5–7)	539	7 (6–8)	840	6 (5–7)	683	6 (5–7)	357	7 (6–8)
**CATASTROPHIZING**												
**PCS**	2846		435		535		840		679		357	
• Total score												
Median, quartiles		29 (21–37)		22 (13–30)		21 (14–30)		31 (25–38)		33 (25–41)		35 (27–43)
Mean, *SD*		30 (11.9)		22 (10.8)		22 (10.9)		32 (9.6)		34 (10.6)		36 (11.2)

**Table 2:** **Comparison of different cut-off point (CP) schemes for classifying Numeric Rating Scale (NRS) scores as mild, moderate or severe pain in terms of interference with functioning: ***F***-value in ANOVA using the CP scheme as independent variable and the Pain Disability Index (PDI) scores as dependent variables, for all patients and for the subgroups with low and high catastrophizing tendency (i.e., Pain Catastrophizing Scale (PCS) scores ≤ or > the median of the scores, 29)**.

	**CP 3,6**	**CP 3,7**	**CP 4,5**	**CP 4,6**	**CP 4,7**	**CP 4,8**	**CP 5,6**	**CP 5,7**	**CP 5,8**	**CP 5,9**	**CP 6,7**
**ALL PATIENTS (*N* = 2854)**											
CP scheme–PDI	332.63	306.15	317.17	337.60	334.67	253.48	337.63	369.65	324.08	306.96	291.35
Ranking	5			3	4		2	1	6		
**PATIENTS WITH PCS TOTAL SCORE ≤ 29 (*N* = 1461)**											
CP scheme–PDI	173.14	140.30	163.10	172.20	152.79	122.50	170.00	172.34	157.38	154.46	136.35
Ranking	1		5	3			4	2	6		
**PATIENTS WITH PCS TOTAL SCORE > 29 (*N* = 1385)**											
CP scheme–PDI	124,57	129.58	121.62	130.02	143.46	101.41	132.35	156.76	130.55	121.63	123.61
Ranking		6		5	2		3	1	4		

*Rankings are given below the F-values, from the highest (1) to the lowest (6) rank*.

*CP: cut-off points, figures refer to highest scores in the first and second categories, for example CP 4,7 means a CP scheme where the first category includes the NRS scores 1–4, the second category NRS 5–7 and the third category NRS 8–10*.

Bootstrapping analysis identified the optimal CP scheme (5,7) in 90.2% of the bootstrapping samples. The 3,6 scheme was identified as the optimal CP scheme in 3.4% of the samples and the 4,6 scheme in 3.3%.

The patients' sex did not influence the optimal CP scheme: in the analyses in which sex and the interaction variable sex^*^CP scheme were entered as co-variates, neither of these covariates contributed significantly to the model. In the analyses in which the PCS score and the interaction variable PCS score^*^CP scheme were entered as co-variates in catastrophizing, the PCS score contributed significantly to the model in all analyses, while the interaction variable PCS score^*^CP scheme contributed sometimes (i.e., in 2 of the 28 analyses). The latter finding was explained as chance variation because only 2 of the analyses found a significant contribution. To explore the finding of the significant contribution of the PCS scores to the models, we conducted more analyses, as described above. First we split the total group into patients with low and with high catastrophizing tendency, and since the median of the PCS score was 29, we performed the analyses separately for patients with a PCS score equal or lower than 29 and for those with a PCS score higher than 29. For the patients with low catastrophizing tendency, i.e., a PCS score ≤ 29, the optimal CP scheme proved to be 3,6 and for the patients with high catastrophizing tendency, i.e., a PCS score > 29, the optimal CP scheme was 5,7 (see Table 2). In the subgroup with low catastrophizing tendency, bootstrapping analysis identified the optimal CP scheme as 3,6 in 29% of the bootstrapping samples, while the 5,7 scheme was identified as the optimal CP scheme in 23% of the samples and the 4,6 scheme in 21%. In the subgroup with high catastrophizing tendency, bootstrapping analysis identified the optimal CP scheme as 5,7 in 87% of the bootstrapping samples, while the 4,7 scheme was identified as the optimal CP scheme in 11% of the samples and the 4,6 scheme in 10%.

Secondly, we split the total group into patients with low, moderate, and high catastrophizing tendencies, and since the lower quartile of the PCS score was below 21 and the higher quartile was above 37, we performed the analyses separately for patients with a PCS score equal to or lower than 21, for PCS scores between 21 and 37, and for those with a PCS score higher than 37. For the patients with low catastrophizing tendency, i.e., a PCS score ≤ 21, the optimal CP scheme proved to be 3,6. For the patients with moderate catastrophizing tendency, i.e., > 21 and ≤ 37, and for those with high catastrophizing tendency, i.e., a PCS score > 37, the optimal CP scheme was 5,7 in both cases. In the subgroup with low catastrophizing tendency, bootstrap analysis identified the optimal CP scheme as 3,6 in 42% of the bootstrapping samples, while the 4,6 scheme was identified as the optimal CP scheme in 19% of the samples and the 5,7 scheme in 18%. In the subgroup with moderate catastrophizing tendency, bootstrapping analysis identified the optimal CP scheme as 5,7 in 87% of the bootstrapping samples, while the 4,6 scheme was identified as the optimal CP scheme in 3% of the samples and the 4,7 scheme also in 3%. In the subgroup with high catastrophizing tendency, bootstrapping analysis identified the optimal CP scheme as 5,7 in 35% of the bootstrapping samples, while the 2,6 scheme was identified as the optimal CP scheme in 22% of the samples and the 2,5 scheme in 12%.

## Discussion

The aim of the current study was to find the optimal cut-off points for mild, moderate, and severe pain in terms of pain-related interference with functioning in patients with chronic musculoskeletal pain, as well as to measure the variability of the optimal cut-off points and determine the association between these cut-off points and patients' catastrophizing tendency and sex. The NRS score cut-off points (CPs) of 5 and 7 (i.e., a 5,7 CP scheme) were found to provide the best model fit, indicating that an NRS score ≤ 5 corresponds to mild interference of pain with functioning, 6 and 7 to moderate interference and 8–10 to severe interference. The variability of the optimal CP scheme was low, as bootstrapping found the 5,7 CP scheme to be optimal in ~90% of the samples. This makes it unlikely that our findings were due to chance fluctuations.

No clear association was found between the cut-off points and patients' sex. In clinical practice, therefore, interpreting the NRS as mild, moderate or severe pain in terms of interference with functioning is independent of the patient's sex. By contrast, the level of catastrophizing influenced the optimal CP scheme: the optimal scheme for patients with low catastrophizing tendency was 3,6, indicating that an NRS score ≤ 3 corresponds to mild interference of pain with functioning, 4–6 to moderate interference, and 7–10 to severe interference, whereas the optimal scheme for patients with high catastrophizing tendency was the same as for the total patient sample, i.e., 5,7, indicating that an NRS score ≤ 5 corresponds to mild interference of pain with functioning, 6 and 7 to moderate interference and 8–10 to severe interference. In terms of the cut-off points between mild and moderate, this finding implies the following: among patients with low catastrophizing tendency, the interpretation of an NRS score of 4 or 5 is that the patients with these scores experience moderate interference of their pain with functioning, while among patients with high catastrophizing tendency, the interpretation of the NRS score 4 or 5 is that the patients with these scores experience mild interference of their pain with functioning.

**Figure 1:**
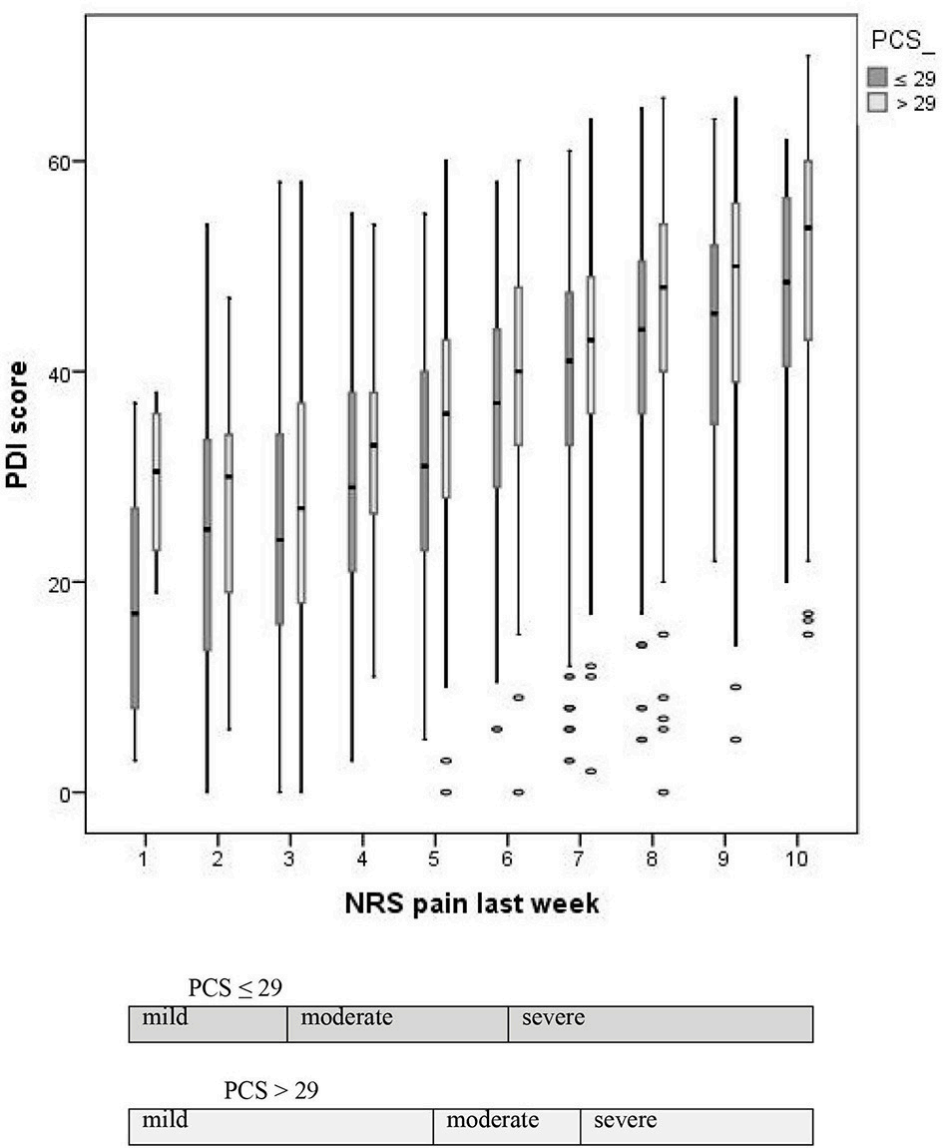
**Boxplots of the Pain Disability Index (PDI) scores by Numeric Rating Scale (NRS) score for average pain during the last week for the patients with low and high catastrophizing tendency (i.e., lower or higher than the median of the total scorer on the Pain Catastrophizing Scale (PCS), viz. 29)**.

Moderate interference with functioning would theoretically imply a higher PDI score than mild interference. However, as can be seen in Figure 1, the PDI scores of the patients with low catastrophizing tendency were lower for each NRS score than those of the patients with high catastrophizing tendency, thus including the group of patients with NRS scores 4 and 5. This contradicts the cut-off point schemes and their interpretation. Two possible explanations may be given. Firstly, the optimal CP scheme for patients with a low catastrophizing tendency may actually also be 5,7 and our finding of the 3,6 scheme was a matter of chance variability. In the subgroup with lower catastrophizing tendency (both the subgroup with a PCS score lower than the median and the subgroup with PCS scores in the lower quartile), the variability was much higher than in the subgroup with higher catastrophizing tendency. The probability that the correct optimal CP scheme was not found is therefore rather high (type 1 error). Secondly, the statistical method introduced by Serlin et al. (1995), which uses the highest *F*-value to indicate the classification scheme that maximizes the differences between the groups and is therefore the most useful for distinguishing between mild, moderate, and severe pain-related interference, may not be the best method for finding the optimal CP scheme.

Optimal cut-off points of 5 and 7 were only mentioned in the literature by Zelman et al. (2003), for patients with osteoarthritis. That this particular CP scheme was found in only one other study may be due to the fact that it was not assessed by most other authors (see Table 3). Our previous study (Boonstra et al., 2014) in a comparable population (not including patients of the present study), but with a smaller sample, found 3 and 6 to be the optimal cut-off points between mild, moderate, and severe interference with functioning, whereas the present study found this 3,6 scheme to be only the fifth best CP scheme. Our previous study used domains of the SF-36 (Aaronson et al., 1998) to measure interference with functioning, and the Visual Analogue Scale (VAS) for pain, instead of the PDI and NRS, respectively. These different measures may be the reason why we found a different CP scheme in the present study. Other reasons may be chance variability and a possible difference in the distribution of the PCS scores, as the CP scheme is the same as that found in the subgroup of patients with low catastrophizing tendency.

**Table 3:** **Published studies about optimal cut-off point schemes for mild, moderate, and severe pain in terms of interference with functioning**.

**Study, authors**	**Type of pain/diagnosis**	**Pain measurement**	***n***	**Optimal cut-off points found in the study**		**Range of values studied by the authors for the lower cut-off point (between mild and moderate), and the higher cut-off point (between moderate and severe)**
				**Lower**	**Higher**	
Serlin et al., 1995	Cancer pain	NRS, worst pain	470	4	6	Lower cut-off point: 3–4
						Higher cut-off point: 6–7
Jensen et al., 1999	Leg amputation patients:	NRS, average pain				
	Phantom pain		74	4	7	Lower cut-off point: 3–4
	Back pain		29	4	6	Higher cut-off point: 6–7
	General pain		102	3	6	
Zelman et al., 2003	Low back pain	NRS, average pain	96	5	8	Lower cut-off point: 4–6
	Osteoarthritis		98	5	7	Higher cut-off point: 6–8
Turner et al., 2004	CTS	NRS, average pain		No superior		Lower cut-off point: 3–5
	Low back injuries			scheme	6	Higher cut-off point: 6–7
				4		
Zelman et al., 2005	Diabetic peripheral neuropathy	NRS, worst and average pain	255	4	7	Lower cut-off point: 4–6
						Higher cut-off point: 6–8
Paul et al., 2005	Cancer pain	NRS, average pain	160	4	7	Lower cut-off point: 3–5
						Higher cut-off point: 5–7
Fejer et al., 2005	Neck pain	NRS, average, worst, and characteristic pain	1385	4	7	14 categories between 3 and 8
Hanley et al., 2006	Spinal cord injury	NRS, (a) overall pain or (b) current pain at worst location	a: 307 b: 174	a and b: 3	a: 7	Lower cut-off point: 3–4
					b: 6	Higher cut-off point: 6–7
Li et al., 2007	Cancer pain, patients with bone metastases	NRS, (a) worst, (b) average, and (c) current	199	a and b: 4, c: 2	a, b, and c: 6	Lower cut-off point: 2–8
						Higher cut-off point: 3–9
Kapstad et al., 2008	Osteoarthritis of the hip	NRS, average pain	224	4	6	Lower cut-off point: 3–5
	Osteoarthritis of the knee		94	4	7	Higher cut-off point: 5–7
Kalyadina et al., 2008	Cancer pain, hematological malignancies or solid tumors	NRS, worst pain	221	4	6	Lower cut-off point: 3–4
						Higher cut-off point: 6–7
Ferreira et al., 2011	Cancer pain	NRS, worst pain	143	4	7	Lower cut-off point: 3–5
						Higher cut-off point: 5–7
Hoffman et al., 2010	Diabetic peripheral neuropathy	NRS, average pain	401	3	6	Not mentioned
Hirschfeld and Zernikow, 2013	Children and adolescents with chronic pain	NRS, maximum pain				Lower cut-off point: 2–7
						Higher cut-off point: 3–8
	Whole sample		2249	4	8	
	Constant pain		650	5	8	
	Chronic headache		430	4	8	
	Musculoskeletal pain		295	2	8	
Boonstra et al., 2014	Musculoskeletal pain	VAS, average pain	456	3	6	Lower cut-off point: 3–5
						Higher cut-off point: 5–7
Brailo and Zakrzewska, 2015	Nondental orofacial pain	NRS, average pain	245	4	7	Lower cut-off point: 3–5
						Higher cut-off point: 5–9
Present study	Musculoskeletal pain	NRS, average pain	2854	5	7	Lower cut-off point: 2–8
						Higher cut-off point: 3–9

*Cut-off points (CP): figures refer to highest scores in the first and second categories, for example CP lower 4, higher 7 means: first category includes the NRS scores 1–4, second category NRS 5–7, third category NRS 8–10*.

*NRS: numeric rating scale; VAS: visual analog scale*.

The association between catastrophizing and cut-off points has not been studied before, so no comparison with other studies is possible. As far as we are aware, only Fejer et al. (2005) studied the influence of patients' sex on the cut-off points for interference with functioning, and their analysis of CP schemes for average pain found a small difference between the sexes, viz. a lower cut-off point between mild and moderate pain interference for women (4) than for men (6). Their other analyses, with the worst and what they called characteristic pain as independent variables, found no or other differences between women and men, and they finally concluded that the differences were small.

The main strength of our study was the large study sample, the largest sample used until now in studies of this topic. It was also the first study taking patient's catastrophizing into account and the second to examine the influence of sex on the CP schemes.

## Limitations

One weakness of our study is the way the patients were included, i.e., using data from a nationwide survey, which meant that response rate and hence selection bias were unknown. In some rehabilitation centers, the localization of pain complaints was not recorded in the survey questionnaire for most patients (see Table 1). Moreover, none of the rehabilitation centers comprehensively recorded the diagnoses in the survey.

Secondly, our study used the PDI to measure interference with functioning. It is possible that other instruments, such as the BPI, would have given different results. Finally, we explored the effect of catastrophizing by splitting the population using the median split and quartile split methods. Although these are common methods to split a population, they may have influenced the results.

### Conclusion

In conclusion, we found that NRS scores ≤ 5 correspond to mild pain-related interference with functioning, scores of 6 and 7 to moderate interference and scores ≥8 to severe interference. This interpretation of the NRS in terms of mild, moderate and severe interference with functioning is independent of the patient's sex, but seems to be influenced by their catastrophizing tendency. However, the difference in CP schemes we found for patients with lower and higher catastrophizing tendencies contradicts what is theoretically plausible. The reason why we did not find the same optimal CP scheme in the subgroups of patients with lower and higher catastrophizing tendencies may be chance variability.

## Author contributions

AB, contributed to the design of the work; and the acquisition, analysis, and interpretation of data; drafted the work, approves final version to be published; agrees to be accountable for all aspects of the work in ensuring that questions related to the accuracy or integrity of any part of the work are appropriately investigated and resolved. RS contributed to design of the work; analysis, and interpretation of data for the work; revised the work critically for important intellectual content; approved final version to be published; agrees to be accountable for all aspects of the work in ensuring that questions related to the accuracy or integrity of any part of the work are appropriately investigated and resolved. HS, AK, RO, JS, KS, contributed to design of the work; and interpretation of data for the work; revised the work critically for important intellectual content; approved final version to be published; and agrees to be accountable for all aspects of the work in ensuring that questions related to the accuracy or integrity of any part of the work are appropriately investigated and resolved.

### Conflict of interest statement

The authors declare that the research was conducted in the absence of any commercial or financial relationships that could be construed as a potential conflict of interest.
